# Heterochromatin Is Not the Only Place for satDNAs: The High Diversity of satDNAs in the Euchromatin of the Beetle *Chrysolina americana* (Coleoptera, Chrysomelidae)

**DOI:** 10.3390/genes15040395

**Published:** 2024-03-22

**Authors:** José M. Rico-Porras, Pablo Mora, Teresa Palomeque, Eugenia E. Montiel, Diogo C. Cabral-de-Mello, Pedro Lorite

**Affiliations:** 1 Department of Experimental Biology, Genetics Area, University of Jaén, Paraje las Lagunillas s/n, 23071 Jaén, Spain; jmrico@ujaen.es (J.M.R.-P.); pmora@ujaen.es (P.M.); tpalome@ujaen.es (T.P.); 2 Department of Biology, Genetics, Faculty of Sciences, Autonomous University of Madrid, 28049 Madrid, Spain; eugenia.montiel@uam.es; 3 Center for Research in Biodiversity and Global Change, Autonomous University of Madrid, 28049 Madrid, Spain; 4 Department of General and Applied Biology, Institute of Biosciences/IB, UNESP—São Paulo State University, Rio Claro 13506-900, SP, Brazil; cabral.mello@unesp.br

**Keywords:** Chrysomelidae, karyotype, repetitive DNA, satellite DNA, satellitome, fluorescence in situ hybridization, genome evolution, heterochromatin, euchromatin, chromosome in silico mapping, CHRISMAPP

## Abstract

The satellitome of the beetle *Chrysolina americana* Linneo, 1758 has been characterized through chromosomal analysis, genomic sequencing, and bioinformatics tools. C-banding reveals the presence of constitutive heterochromatin blocks enriched in A+T content, primarily located in pericentromeric regions. Furthermore, a comprehensive satellitome analysis unveils the extensive diversity of satellite DNA families within the genome of *C. americana*. Using fluorescence in situ hybridization techniques and the innovative CHRISMAPP approach, we precisely map the localization of satDNA families on assembled chromosomes, providing insights into their organization and distribution patterns. Among the 165 identified satDNA families, only three of them exhibit a remarkable amplification and accumulation, forming large blocks predominantly in pericentromeric regions. In contrast, the remaining, less abundant satDNA families are dispersed throughout euchromatic regions, challenging the traditional association of satDNA with heterochromatin. Overall, our findings underscore the complexity of repetitive DNA elements in the genome of *C. americana* and emphasize the need for further exploration to elucidate their functional significance and evolutionary implications.

## 1. Introduction

The Chrysomelidae family, known as leaf beetles, belongs to the Polyphaga suborder and is considered one of the most diverse beetle (Coleoptera) families [1]. Chrysomelids are phytophagous (both in the larval and adult phases), most of which are monophagous or oligophagous. The genus *Chrysolina* comprises more than 400 species, grouped into 65 subgenera, distributed throughout the world, except for in South America, Australia, and Antarctica [2]. *C. americana* feeds on the leaves of plants from the Lamiaceae family, such as rosemary, lavender, or thyme, among others. It is considered native to the Mediterranean, present in several European countries, and in North Africa [3,4]. However, it has currently spread to Northern Europe, becoming an invasive species in some of these countries, as well as in Israel and Cyprus [5,6].

A large percentage of the genome in most eukaryotic species is composed of sequences repeated hundreds or thousands of times [7]. The quantity, types, and distribution of these repetitive DNAs influence the structure, size, and diversity of genomes [8]. Repetitive DNA consists of the following two main types of repeats: dispersed repeats and tandem repeats. Dispersed repeats are known as transposable elements (TEs), while tandem repeats are mainly referred to as satellite DNA (satDNA). Satellite DNA has long been considered as ‘junk’ DNA, because it does not encode proteins and, thus, it seemed to lack biological function [9]. However, data obtained in recent decades have challenged this view. It has evidenced satDNAs as a significant contributor to chromosome architecture, function, and evolution [10,11,12,13]. In general, centromeres are composed of satDNA sequences, which contribute to the assembly processes of centromeric chromatin [14,15]. Satellite DNA plays a role in chromosome segregation during mitosis, meiosis, and segregation distortion and its evolution can trigger reproductive isolation and ultimately speciation [16,17,18,19]. Additionally, pericentromeric satDNA contributes to a higher level of nuclear organization and the maintenance of genome integrity [20]. Beyond its structural role in the genome, satDNA transcription has a functional importance, such as in the formation and maintenance of heterochromatin, defining the centromere identity, and preserving genome stability (reviewed in [21,22]). Misregulation of satDNA expression can adversely affect genomic architecture, chromosome segregation, and gametogenesis. Changes in satDNA copy number and transcription rates may be associated with stress, environmental adaptations, and pathological states such as oncogenic transformation [10,23,24,25,26,27]. Among insects, the transcription of satDNA has been described in several orders (see [28] and references therein).

Currently, the study of satDNA in insects has advanced due to the affordability of next-generation sequencing (NGS) techniques. Massive sequencing combined with subsequent bioinformatics analysis is now commonly used for satDNA investigation [29,30]. The bioinformatics analysis of satDNA from NGS data has been made possible by the development of specialized pipelines that can identify repeated sequences in the genome, without the need for assembly or a reference genome, using the programs RepeatExplorer [31,32], along with the TAndem REpeat ANalyzer (TAREAN) [33] (https://repeatexplorer-elixir.cerit-sc.cz, accessed on 4 December 2023). This methodology for satDNA analysis has allowed the identification of a plentiful amount of satDNAs that are termed ‘satellitome’, which refers to the whole catalogue of satDNA families in a given species [34]. Among insects, these tools have been successfully applied for satDNA analysis in various species; but, within the Coleoptera order, this methodology has only been applied to the following five species: *Hippodamia variegata* Goeze, 1777 (Coccinellidae) [35]; *Rynchophorus ferrugineus* Olivier, 1790 (Curculionidae) [28]; *Rhyzopertha dominica* Fabricius, 1792 (Bostrichidae) [36]; *Tribolium castaneum* Herbst, 1797 (Tenebrionidae) [37]; and *Tenebrio molitor* Linnaeus, 1758 (Tenebrionidae) [38].

Among chrysomelid beetles, there is no description of satellitomes, although the group is an interesting source for study, as the karyotypes are highly variable in the family, along with the variation of heterochromatin [39,40]. In Polyphaga beetles, the three highest rates of karyotype evolution were observed in the Chrysomelidae family, mainly in the genera *Cyrtonus*, *Chrysolina*, and *Timarcha* [40,41]. Studies on satDNA in Chrysomelidae are limited, since only four species have been analyzed, as follows: *Leptinotarsa decemlineata* Say, 1824; *Xanthogaleruca luteola* Müller, 1766; *Chrysolina carnifex* Fabricius, 1792; and *C. americana* [42,43,44,45]. In these species, classical techniques such as restriction enzyme digestion have been used to isolate satDNA, enabling the characterization of only one or two satDNA families per species. Fluorescent in situ hybridization (FISH) of these families evidenced these sequences as a component of the pericentromeric regions of the chromosomes [42,43,44,45]. In order to advance on the understanding of satDNAs in beetles, in this work, we applied genome sequencing along with bioinformatic and chromosomal analysis to characterize the satellitome of the chrysomelid beetle *C. americana*. We utilized a chromosome-scale genome assembly of *C. americana* to map all satDNA families. We have termed this procedure CHRISMAPP (CHRomosome In Silico MAPPing), thereby emphasizing the significance of chromosome mapping as the aim of this technique. Besides the detailed characterization of satDNAs in the species, our study reveals that a high diversity of satDNA families could be placed on euchromatin, being the heterochromatic region composed only of a few families of highly amplified satDNA. These data add important information on the general ideas about satDNA organization and evolution.

## 2. Material and Methods

### 2.1. Insects, Preparation of Chromosome Spreads, and C-Banding

Male specimens of the species *C. americana* were collected at the campus of the University of Jaén (Spain). As *C. americana* is not an endangered species, no special permission was required. Males were dissected under a binocular microscope to extract their testes. The extracted testes were then immersed in distilled water for 45 min to induce an osmotic shock. Subsequently, the distilled water was removed and a Carnoy fixative solution of ethanol and glacial acetic acid (3:1) was added. The samples were stored in the freezer at −20 °C until use. For slide preparation, a testicular lobe was taken in 25 µL of 50% acetic acid and disaggregated using a micropipette. The resulting material was deposited as 4 or 6 drops onto a preheated slide on a heating plate set at 42 °C and was stained with Giemsa. The C-banding technique used for chromosome preparations is based on the method described by Sumner [46], with small modifications. The chromosome preparations were initially treated with a 0.2 N hydrochloric acid solution for 1 min at room temperature. Subsequently, they were incubated in a 5% barium hydroxide solution at 60 °C for 1 min and 30 s. The preparations were then washed with water, followed by a brief rinse in the first hydrochloric acid solution and a final wash in 2× SSC at 60 °C for 2 min. The preparations were stained with Giemsa or with 4′-6-diamino-2-phenylindole (DAPI) (Roche, Applied Science, Basel, Switzerland), at a concentration of 0.75 µg/mL.

### 2.2. DNA Extraction, Genome Sequencing, and Bioinformatic Analysis

Genomic DNA (gDNA) extraction was carried out from one adult male using the NucleoSpin Tissue kit (Macherey-Nagel GmbH & Co., Düren, Germany), following the provider’s instructions. Three micrograms of genomic DNA were submitted to the company Novogene for sequencing on the Illumina HiSeq 2000 platform (San Diego, CA, USA). A 350 bp fragment library and 101 bp paired-end sequencing reads were obtained, providing a total of 2 Gb of sequencing data. The quality of the raw reads was assessed using the FastQC program [47]. Reads with a quality score below 20 and those containing adapters were filtered out using Trimmomatic v0.39 [48]. After filtering, a file was generated containing 12 million randomly selected reads (6 million paired-end reads). This file was then uploaded to the Galaxy platform (https://galaxy-elixir.cerit-sc.cz/, accessed on 4 December 2023) and processed using RepeatExplorer2 v2.3.7 [31] for the analysis and characterization of repetitive DNA sequences. Default options were chosen, except for the threshold of the genome percentage used to define a ‘top cluster’, which was set to 0.001%. In order to identify the specific clusters that contain satDNA sequences, and to determine the size of the repeat unit (or monomer), as well as the consensus sequence for each family, Geneious v4.8.5 [49] was utilized. The satDNA families were named following a nomenclature similar to that proposed by Ruiz-Ruano et al. [34]. The naming convention includes the first letter of the genus name; the first three letters of the species name, followed by ‘Sat’; a number indicating the families in order of abundance; and a number representing the monomer size for each satDNA family. The quantification and divergence values of each satDNA family were calculated using RepeatMasker v4.1.4. This involved aligning a file containing 5 million randomly selected reads with the complete library of satDNA dimers (for families with a repeat unit length greater than 100 bp) or a concatenation of monomers comprising of at least 200 bp for families with a repeat unit length less than 100 bp.

### 2.3. Fluorescence In Situ Hybridization (FISH) and Chromosome In Silico Mapping (CHRISMAPP)

For the chromosomal localization of the most abundant satDNA families, specific oligonucleotides were designed based on the most conserved regions of the repeat unit sequences (Table 1). These oligonucleotides were directly labeled with either biotin-16-dUTP (Roche) or digoxigenin-11-dUTP (Roche) using terminal transferase (Roche) and following the instructions provided by the supplier.

A double-stranded telomeric TTAGG probe was generated through PCR, using the primers (TTAGG)_6_ and (TAACC)_6_ without template, following a similar procedure as described by Ijdo et al. [50]. The PCR product (1 µg) was labeled with biotin-16-dUTP using the Biotin-Nick Translation Mix (Roche).

Fluorescence in situ hybridization (FISH) was performed following the protocol of Cabral-de-Mello and Marec [51], with some modifications. In total, 20 μL of the hybridization solution (50% deionized formamide (*v*/*v*), 10% dextran sulfate (*v*/*v*), and 2× SSC), containing 3 pmol/µL of the satDNA oligonucleotide probe or 10 ng/µL of the telomere probe, was administered per slide. For two-color FISH experiments, 40 μL of the hybridization solution with 3 pmol/µL for each probe was used, one labeled with biotin-16-dUTP and the other with digoxigenin-11-dUTP. The slides were subjected to incubation on a heated surface at a temperature of 70 °C for a duration of 2 min and 30 s, transferred to a humid chamber, and subjected to overnight incubation (16–18 h) at a temperature of 37 °C. Immunological detection was performed using either streptavidin conjugated with Alexa Fluor™ 488 (Thermo Fisher Scientific, Waltham, MA, USA) at a concentration of 10 μg/mL or anti-digoxigenin-rhodamine (Roche) at a concentration of 1 μg/mL, depending on whether the probes were labeled with biotin-16-dUTP or digoxigenin-11-dUTP, respectively.

The preparations were mounted with VECTASHIELD with DAPI (Vector Labs, Burlingame, CA, USA). The mounted preparations were observed under an Olympus BX51 fluorescence microscope (Olympus, Hamburg, Germany) equipped with an Olympus DP70 camera and appropriate filters. Image acquisition and processing were carried out using DP Manager software v1.1.1.71 and Adobe Photoshop CS4 software (Adobe Systems, San Jose, CA, USA).

For the amplification of hybridization signals from the CameSat004-10 and CameSat005-322 satDNA families, which did not show visible hybridization signals with the initial protocol, the avidin-FITC/antiavidin-biotin system [52] was utilized, with one or two rounds of amplification. Before the immunological detection, slides were incubated in the washing blocking buffer (WBB) (4× SSC, 0.1% *v*/*v* Tween 20, and 1% *w*/*v* skimmed milk) for 30 min. Chromosome preparations were then incubated with avidin-FITC (Vector Labs) (5 µg/mL in WBB) for 30 min, followed by three washes of 5 min with WBB. Subsequently, preparations were incubated for 30 min with the biotinylated anti-avidin solution (5 µg/mL in WBB), followed by three washes of 5 min with WBB. This process was repeated with another incubation with avidin-FITC, followed by three washes for 5 min with WBB, before being air-dried and mounted with VECTASHIELD with DAPI (Vector Labs).

We used the chromosome-scale genome assembly of *C. americana* (https://www.ncbi.nlm.nih.gov/datasets/genome/GCA_958502065.1/, accessed on 2 February 2024) to map the satDNA families through a new simplified approach based in the pipeline of Tunjić-Cvitanić et al. [53], which we have designated as CHRISMAPP (CHRromosome In Silico MAPPing). Initially, the consensus sequence of each satDNA family was annotated in the chromosome-scale genome assembly utilizing Geneious 2023.2.1 [49], employing a similarity threshold of 70%. Subsequently, the resultant .gff file, containing comprehensive annotations, underwent processing in Rstudio [54], using the ggplot2 package [55]. A bespoke script was implemented to visualize the previously acquired annotations. This script is included in the Supplementary Materials.

## 3. Results and Discussion

### 3.1. General Features of Chromosomes of C. americana

**Figure 1:**
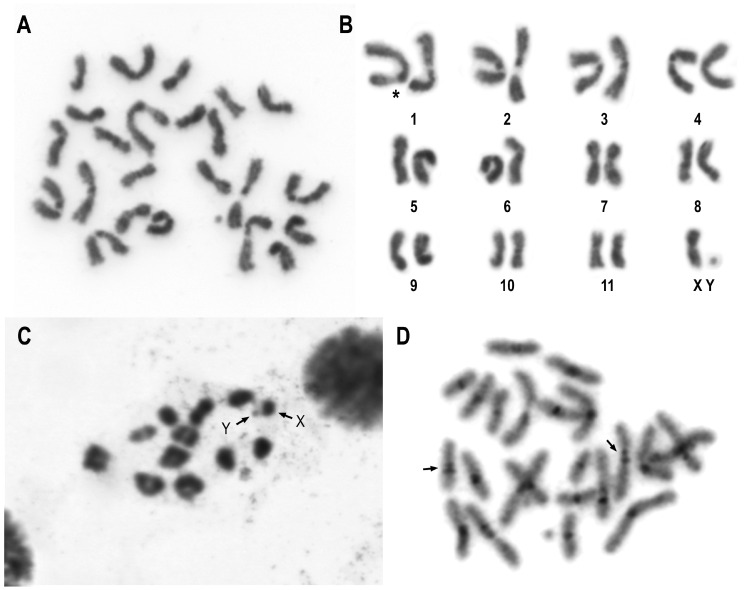
(**A**) Male mitotic metaphase and (**B**) karyotype of *C. americana*. The karyotype consists of 11 pairs of autosomes and the sex pair, a metacentric X chromosome and a dot-shaped Y chromosome. Pair one exhibits significant heteromorphism due to the size of the secondary constriction located on the long arm (*). (**C**) Meiotic metaphase with 11 autosomal bivalents and the sex chromosomes in an Xy_p_ “parachute” shape. (**D**) Mitotic metaphase after the C-banding technique and subsequent DAPI staining (inverted image). Heterochromatin blocks are observed in the pericentromeric regions of all chromosomes except for the Y chromosome. Arrows indicate some chromosomes displaying two heterochromatin blocks.

*C. americana* shows a karyotype of 2n = 22 + XX in females and 2n = 22 + Xy_p_ in males (Figure 1A,B). The karyotype observed in this study aligns with the karyotype described by Petitpierre [56]. All autosomes display a metacentric morphology, except for one pair that is submetacentric. According to Petitpierre [56], the submetacentric chromosomes correspond to the fifth largest pair of autosomes. In our study, we have numbered the submetacentric pair as pair number 6 based on the results of the FISH, as will be discussed later. The X chromosome also appears to be metacentric. The Y chromosome, due to its diminutive size, cannot be morphologically characterized. Various autosomal pairs exhibit secondary constrictions. The largest pair of autosomes shows a pronounced heteromorphism due to a secondary constriction in its long arm. Species within the Chrysomelidae family possess varying chromosomal numbers, ranging from 2n = 8 to 2n = 72, with 2n = 24 being the most prevalent [40,57]. During metaphase I, the sex chromosomes are observed to form the typical “parachute” structure (Xy_p_) (Figure 1C), which is a common feature and considered an ancestral character in beetles [58]. This chromosomal system of sex determination is the most frequent among Chrysomelidae beetles (reviewed in [40]).

**Figure 2:**
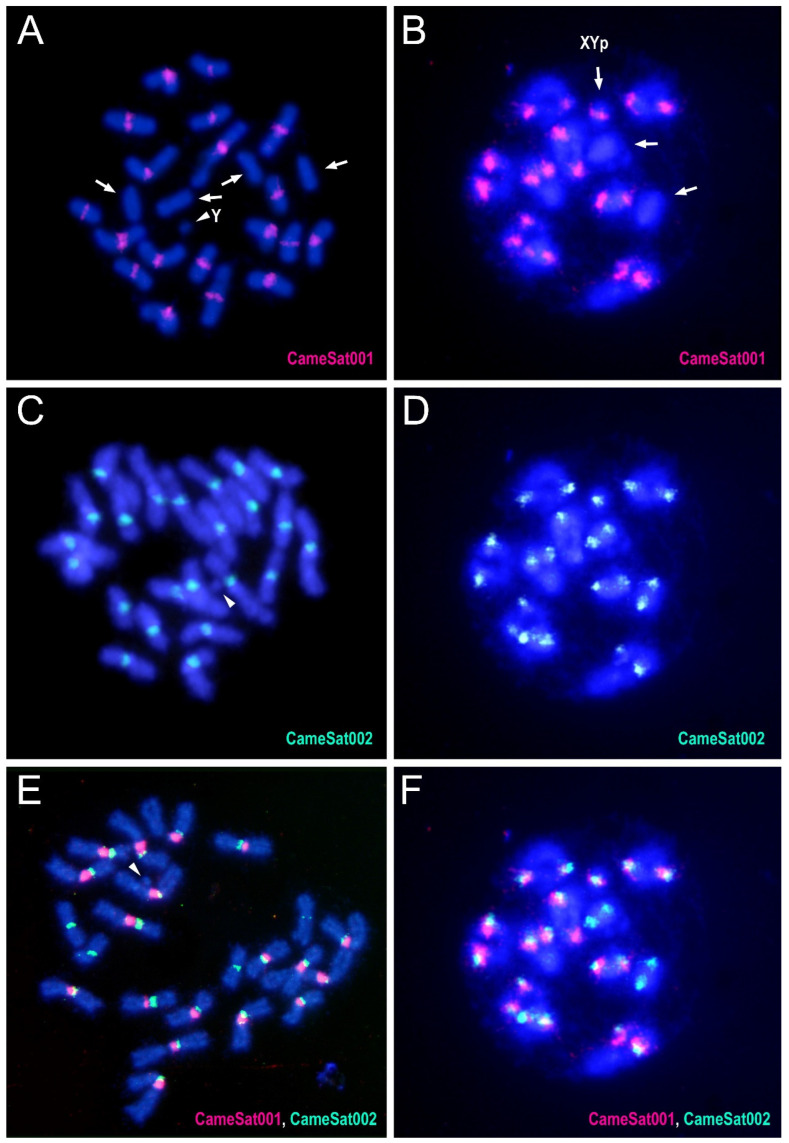
FISH using the CameSat001-141 satDNA (labeled in red) as a probe on both mitotic (**A**) and meiotic (**B**) chromosomes of *C. americana*. Similarly, FISH was conducted using the CameSat002-187 satellite DNA (labeled in green) on mitotic (**C**) and meiotic (**D**) chromosomes. Double FISH using both the probes on mitotic (**E**) and meiotic (**F**) chromosomes. Arrows indicate the chromosomes or the bivalents that did not display a hybridization signal with CameSat001. Arrowheads indicate the Y chromosome, which did not exhibit a hybridization signal with either of the two probes.

The C-banding technique demonstrates the presence of constitutive heterochromatin blocks located in the pericentromeric regions of all chromosomes, except for the Y chromosome, which has no C-heterochromatic positive blocks (Figure 2D). Some autosomes contain two adjacent blocks of heterochromatin in the pericentromeric regions. DAPI staining revealed that the heterochromatic regions are rich in A+T content [42], in a similar manner to that commonly observed on other Coleoptera [59]. Although the number of chrysomelid species analyzed using C-banding is limited, it appears that a shared characteristic among the family is the presence of small pericentromeric blocks of heterochromatin in most autosomes and varying quantities of intercalary heterochromatin on the sex chromosomes [60]. However, in *C. americana* ([42], this study) and in other Chrysomelidae species, substantial amounts of pericentromeric heterochromatin were observed [43,44]. Heterochromatin was particularly abundant in the species *C. carnifex*, where C-banding revealed the existence of large blocks of heterochromatin in all autosomes and both sex chromosomes [45].

### 3.2. Satellitome Analysis of C. americana Genome Reveals Extensive Diversity of satDNAs with Amplification of Few Families

In order to gain insights about the repeat composition of the genomes of beetles, a detailed analysis of the satDNA composition of *C. americana* was performed. The general analysis of 34 million reads of 101 bp revealed enrichment of the genome in A+T content, i.e., 64.17%. Among insects, the genome percentage of A+T content varies from 54.46% in *Buathra laborator* Thunberg, 1822 to 74.26% in *Bombylius discolor* Mikan, 1796 (see [61] for a review) and also in eukaryotes; as a whole, the A+T percentage in genomes has been found to be higher than the C+G percentage [61]. In the same direction, the identified satDNAs were rich in A+T base pairs, also a common feature for beetles and other insect genomes [59]. After the analysis using RepeatExplorer2, we gathered more details about the genome structure of *C. americana*, evidencing the clustering of 3,426,864 reads into 337,707 clusters (Supplementary Figure S1) that collectively constitute approximately 73% of the *C. americana* genome. This analysis provided insights into the number of repetitive sequences present in the genome of this species, evidencing occurrence of satDNAs with distinct abundances, as well as uncharacterized sequences with extremely low levels of repetition. Out of the total number of clusters obtained, 1705 clusters (accounting for 43% of the genome) represent more than 0.001% and are the main genome components, as they present a higher degree of repetition.

Among the 43% of the most abundant genome sequences, a significant supercluster was identified, constituting nearly 9% of the genome. This supercluster corresponds to the most abundant satellite DNA (satDNA) family found in *C. americana*. Additionally, two other superclusters were identified, representing the second and third most abundant satDNA families, which account for approximately 2.5% and 1% of the genome, respectively. For a better characterization of the nearly complete set of satDNAs in the genome (satellitome) of *C. americana*, in addition to the clusters identified by RepeatExplorer as satDNA, other clusters displaying graph patterns with regions of high density, a characteristic feature of satDNA [31], were manually investigated. Through this analysis, the existence of 165 distinct satDNA families was evidenced (Table 2). The abundance of these 165 satDNA families was estimated through RepeatMasker, evidencing that, collectively, the satDNAs account for 17.97% of the *C. americana* genome. On other Coleoptera, the abundance of satDNAs is in a similar place to that observed in *C. americana* and ranges from 11.4% in *R. ferrugineus*, from United Arab Emirates population [28], to 28.2% in *T. molitor* [38].

The three most abundant satDNA families in the genome of *C. americana*, CameSat001-141, CameSat002-187, and CameSat003-10, collectively corresponded to 12.649% abundance. The remaining satDNA families appear with percentages of less than 1% and, altogether, they represented solely about 5.27% of the genome. Among these, some were in really small amounts, representing less than 0.001% of the genome, with CameSat165-105 being the the least abundant, with a genome percentage of 0.0002%. Among the satDNAs identified, the satDNA CameSat002-187 that has considerable abundance (2.41% of abundance) corresponds to the CAMA satDNA family, which was previously described in *C. americana* using isolation through the digestion of genomic DNA with the *Alu*I endonuclease [42] (Supplementary Figure S2). The CameSat035-5-tel family represents the repetitive occurrence of the TTAGG sequence, which is the most prevalent telomeric sequence found in insects [62,63]. FISH demonstrates the terminal location of the TTAGG repeats in the *C. americana* chromosomes (Supplementary Figure S3), as was observed previously [64].

Among Coleoptera species, the satellitomes were better characterized into five species, belonging to three families, with no Chrysomelidae [28,35,36,37,38], revealing a distinct number of satDNA families, from 10 in *R. dominica* [36] to 112 in *R. ferrugineus* [28]. The data from *C. americana* adds information about the huge variation in the number of families of satDNAs on Coleoptera, with this species having the highest number of satDNA families characterized so far, i.e., 165 families. This is also the highest number of satDNA families identified in an insect genome, as there are more than the 160 identified in *Triatoma delpontei* Romaña and Abalos, 1947 [65]. Concerning the abundance of the distinct satDNA families, as observed here in *C. americana*, it is also common for beetles to demonstrate one more abundant satDNA family, as is the case of CameSat001-141 with about 9% abundance. In the other beetle species, the major satDNA represents 26.5% of the genome of *T. molitor* [38], 17% of the genome of *T. castaneum* [37], 9.37% of the genome of *H. variegata* [35], and from 8.43 to 20.45% of the genome of *R. ferrugineus*, depending on the population [28].

Two other characteristics of the satellitome characterized here are the monomer size and divergence of the satDNAs. The repeat unit size of the *C. americana* satDNA families ranged from 5 bp (CameSat035-5-tel) to 3664 bp (CameSat120-3664), with the most common monomer sizes ranging between 101 and 200 bp (Supplementary Figure S4). For a long time, the identification of short or extremely long satDNAs was hampered by the methodology used for satDNA prospection, the restriction enzyme digestion technique. This challenge was overcome by the application of sequencing techniques and bioinformatic tools in the search for satellite DNA families. In this way, the identification of long satDNA, over 1000 bp, has been more common, ranging up to 4228 [66]. Although, in most cases, the satDNAs are shorter, ranging from 100 to 200 bp, as observed here for the majority of the satDNAs in *C. americana*.

The satDNA divergence values for each family, calculated using RepeatMasker and visually represented in Supplementary Figure S5 using Rstudio, vary across the different families, ranging from 1.77% in CameSat074-1625 to 26.18% in CameSat031-281. The average divergence of all satDNA families in *C. americana* is determined to be 9.91%. Satellite DNA divergence is directly influenced by mutation processes and is inversely influenced by amplification and homogenization processes [59,67]. From the analysis of the satDNAs landscape (satDNA abundance versus divergence), it is evident the occurrence of a peak of abundance in K2P divergence of about 5–7% for CameSat001-141 and CameSat002-187, suggesting a more recent amplification or homogenization of these repeats, in comparison to most of the satDNAs identified in the *C. americana* genome. Despite this, the great difference in abundance between CameSat001-141 and CameSat002-187 has peaks in similar K2P divergence that could suggest similar patterns of homogenization. As expected, the homogenization could be a result of the amplification process that is very common for sequences in the pericentromeric region (see [68] for a review).

### 3.3. Characterization and Chromosomal Localization of the Main Families of Satellite DNA Shows an Unpreceded High Number of satDNAs in Euchromatin

As mentioned in the previous section, there are three satellite DNA families that make up more than 1% of the *C. americana* genome. For the two most abundant satDNAs, CameSat001-141 and CameSat002-187, FISH mapping revealed the presence of these satDNAs in the pericentromeric regions of multiple chromosomes. For CameSat001-141, the hybridization signals are located on the pericentromeric regions of all chromosomes, except in the two smaller pairs of autosomes and the Y chromosome (Figure 2A,B). CameSat002-187 was also located in the pericentromeric regions of all chromosomes, except for in the Y chromosome (Figure 2C,D). Notably, both families were found on the X chromosome, but not on the Y chromosome. The satDNA family CameSat002-187 displays a hybridization pattern similar to that described previously for the CAMA satDNA family [41], except regarding the Y chromosome, which shows positive hybridization only for the CAMA satDNA family. The material used in both studies comes from very different geographical locations; the material used by Lorite et al. [41] was collected in Mallorca (Balearic Islands, Spain), while that of this study was collected in the Iberian Peninsula, more specifically in Jaén (Spain). Satellite DNA, as a dynamic component of the genome, can exhibit interpopulation differences, as described in very different species. For example, variation in the chromosomal distribution of satDNA among populations has been described in grasshoppers [69,70]. Variations in satellite DNA among populations have also been described in the fish *Cyprinodon variegatus* Lacepède, 1803 [71] or the beetle *T. castaneum* [72]. More recently, large variations in the chromosomal distribution of the satDNA of the X chromosome have been described in mole species [73]. Interpopulation differences for the amount of satDNA have also been observed from genome sequencing data [28]. Similarly, using genome sequencing data, rapid changes in the quantity and type of repetitive DNAs on the Y chromosome both within and between species of malaria mosquitoes, specifically within the *Anopheles gambiae* Giles, 1902 complex, have been described [74].

**Figure 3:**
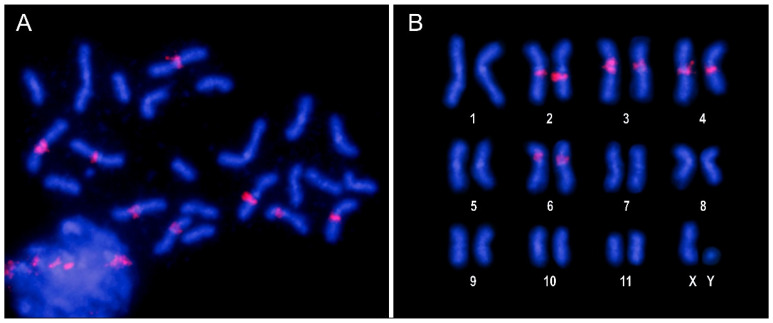
Male mitotic metaphase (**A**) and karyotype (**B**) of *C. americana* following FISH with the CameSat003-10 family, showing the presence of positive hybridization signals on the pericentromeric regions of four pairs of autosomes, including the submetacentric pair 6.

Hybridization analysis for the CameSat003-10 family, which is the third most abundant satellite DNA, reveals a positive hybridization signal exclusively in the pericentromeric regions of four pairs of autosomes (Figure 3). No hybridization signals were observed for the following satDNA families unless signal amplification was performed. The absence of clear hybridization signals may be due to their low abundance in the genome and/or their lack of accumulation in specific regions.

**Figure 4:**
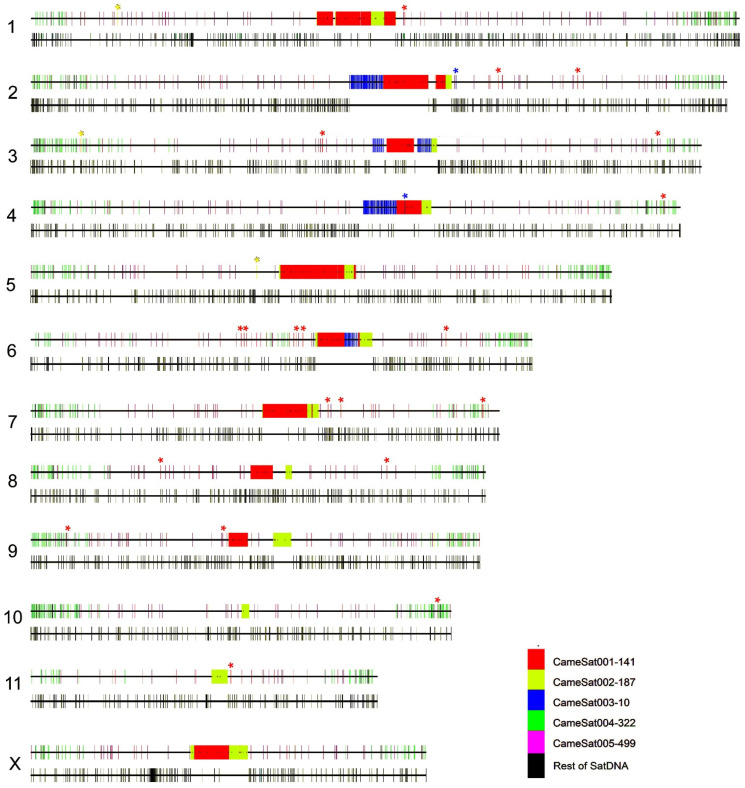
*C. americana* pseudochromosomes showing the distribution of different satDNA families obtained through the CHRISMAPP approach. For each chromosome, two schemes are displayed, the top one illustrates the distribution of the five most abundant satDNA families, while the bottom one shows the distribution of the remaining satDNA families (from CameSat006-2670 to CamaSat165-105). Asterisks indicate the presence of short arrays of the CameSat001-141, CameSat002-187, and CameSat003-10.

The assembled genome of *C. americana* is currently available at the chromosome level, except for the small Y chromosome (GenBank GCA_958502065.1). All satDNA families have been mapped using the sequence of assembled chromosomes via the CHRISMAPP (CHRomosome In Silico MAPPing) approach (Figure 4). To achieve this, satDNA sequences were located within the assembled chromosome sequence and, subsequently, graphically represented. The results obtained with this procedure coincide with those obtained with FISH for the three main families of satellite DNA. This could constitute an alternative method to FISH when material for cytogenetic studies is not available or the sequences are present in a very low amount or do not form long arrays that could hamper the FISH signal visualization. Moreover, it could give more details about the organization of satDNA, like the interspersion of satDNA families and the placement of satDNAs with low abundance in euchromatin.

Following CHRISMAPP, we confirm that the CameSat001-141 satDNA family forms large blocks in the pericentromeric region of most autosomes and the X chromosome but cannot be detected in smaller autosomes (pairs 10 and 11). The CameSat002-197 satDNA family also forms large blocks in the pericentromeric regions, present in all autosomes and the X chromosome. CHRISMAPP results indicate that both satellites do not overlap. FISH, using probes of the two most abundant satellites, demonstrated that although both are located in pericentromeric regions, they appear to form homogeneous blocks and both satellite DNAs are not mixed (Figure 2E,F), as is observed with CHRISMAPP. As observed using FISH, the CHRISMAPP approach shows that the CameSat003-10 satDNA family is present in only four pairs of chromosomes (pairs 2, 3, 4, and 6). On chromosome 3, this satDNA is organized into two different blocks, which is also visible using FISH. As previously indicated, only one of the autosome pairs is submetacentric, the others are metacentric. This chromosome was designated as pair 5 by Petitpierre [56]. FISH with CameSat003-10 shows that the pair of submetacentric chromosomes carries this satDNA. According to the genome assembly of *C. americana*, it corresponds to the sixth largest chromosome, as is presented in the karyotype here (Figure 1); these submetacentric chromosomes have been designated as pair 6. Although most copies of the three main satDNA families are accumulated in blocks in the pericentromeric regions, the CHRISMAPP approach has shown that there are short arrays dispersed throughout the euchromatic regions of the chromosome arms.

**Figure 5:**
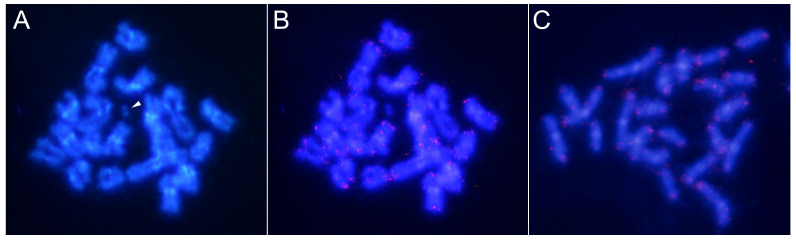
DAPI staining (**A**) and FISH on male mitotic chromosomes of *C. americana* using a probe specific to the CameSat004-322 family with one amplification round (**B**), revealing positive hybridization signals on all chromosomes mainly accumulated at the terminal region or the chromosome arms. (**C**) Hybridization using CameSat004-322 as a probe with two amplification rounds. Arrowhead shows the Y chromosome.

**Figure 6:**
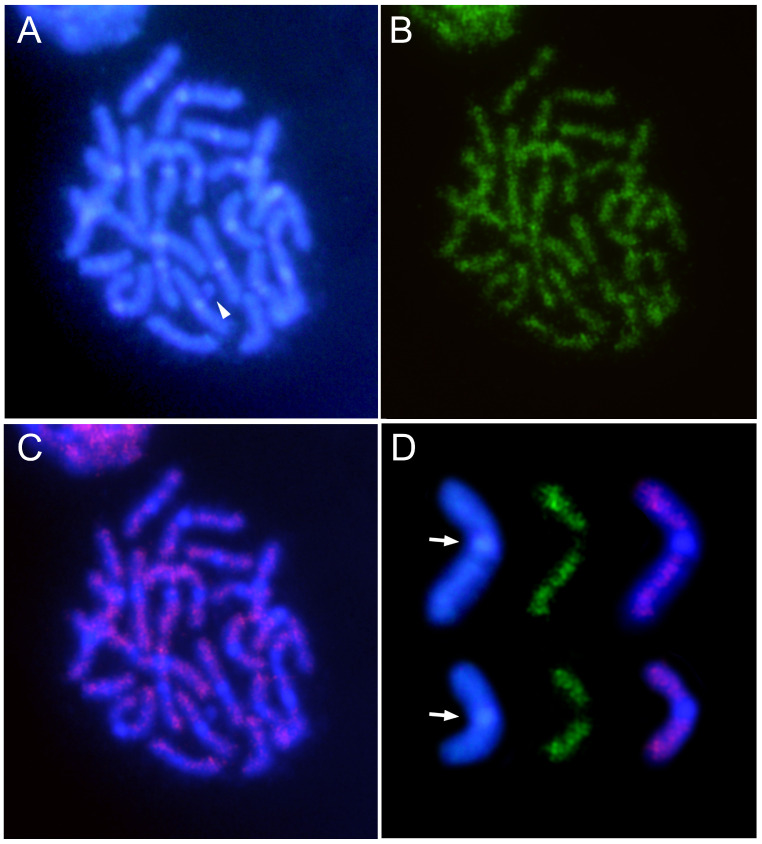
DAPI staining (**A**) and FISH (**B**) on male mitotic chromosomes of *C. americana*, using a specific probe for the CameSat005-499 satDNA family. (**C**) Merged image revealing positive hybridization signals in the euchromatin of all chromosomes. Arrowhead shows the Y chromosome. (**D**) Selected chromosomes after DAPI staining, FISH with the CameSat005-499 satDNA, and merged images. Arrows indicate the DAPI-positive heterochromatic regions that are free of hybridization signals.

The following two more abundant satDNA families (CameSat004-322 and CameSat005-499) appear to be distributed along the chromosome arms, although their distribution patterns are different (Figure 4, Supplementary Figure S6). CameSat005-499 is organized into short arrays that are distributed virtually uniformly along the chromosome arms in all chromosomes, while CameSat004-322 seems to accumulate in the terminal regions of the chromosomes (Figure 4, Supplementary Figure S6). To locate these satDNAs on the chromosomes, FISH was performed using signal amplification. Once again, the results obtained coincide with the results of the CHRISMAPP approach. Using CameSat004-322 satDNA as a probe, hybridization signals were observed dispersed throughout all chromosomes, although they are concentrated in the terminal regions of the chromosome arms (Figure 5). The hybridization pattern obtained with CameSat005-499 is more uniform, with hybridization signals appearing throughout the euchromatin of all chromosomes, while the heterochromatin remained free of hybridization (Figure 6). The Y chromosome shows no hybridization signals with either of these two satDNAs.

Using the CHRISMAPP approach, we have located the remaining 150 families of satDNA (from CameSat006-2670 to CameSat165-105, excluding the family CameSat035-5-tel, which represents telomeric DNA). Together, all these satDNAs form short arrays distributed throughout the genome. In some chromosomes, some of these short arrays are found in pericentromeric regions, interspersed with sequences of the three main satDNAs. However, in some chromosomes, such as pairs 2, 6, and 11, the less abundant satDNAs are mostly located in the euchromatic regions of the chromosome arms.

In summary, the obtained results show that the genome of *Ch. americana* presents a large number of different satDNA families, 165 in total. Of these, only the three most abundant ones are amplified and accumulated in the form of large blocks in pericentromeric heterochromatic regions, while most copies of the remaining satDNA families are dispersed throughout the euchromatic regions. Traditionally, satellite DNA has been predominantly associated with its presence in heterochromatin regions. However, it is now recognized that tandem repeat DNAs, including microsatellites, minisatellites, and satDNAs, can also be found in euchromatin. Recent studies involving the characterization of satellitome and in situ hybridization with low-abundance satDNA families have revealed that many of these satellite DNA sequences are indeed located in euchromatin (see [68,75] for a review). These findings challenge the traditional view and highlight the presence and distribution of satellite DNA in both heterochromatic and euchromatic regions.

## Figures and Tables

**Table 1:** Oligonucleotides used for FISH analyses in the chromosomes of *C. americana*.

SatDNA Family	Oligonucleotide	Sequence
CameSat001-141	Came_CL2-F Came_CL2-R	ACCATGATGCGTGCCAAGTC
		GTCAACAGAAATCATTGTCGGGTC
CameSat002-187	Came_CL26-F Came_CL26-R	ACAAGTCGAGACATACGAAGCAC
		CCAAAATACAGAACAAGTCCAGCTG
CameSat003-10	Came_CL1	GACTTGTCCCGACTTGTCCC
CameSat004-322	Came_CL4-F Came_CL4-R	CGAACGCCAATCGATTCAGAATG
		CAGAATTGCTCTATCTTCAACCGTTC
CameSat005-499	Came_CL11-F Came_CL11-R	ATGTCCGTCGTGGTATTAGCCAG
		ACTCCCAAGCAGCACAGTCTC

**Table 2:** Satellite DNA families identified in *C. americana*, ordered from highest to lowest abundance in the genome. NCBI accession numbers PP502226-PP502390. The K2P (Kimura Two-Parameter model) divergence, the repeat unit length (RUL), and the percentage of A+T are indicated for each family.

satDNA Family	% Genome	Divergence (K2P)	RUL (bp)	A+T (%)	satDNA Family	% Genome	Divergence (K2P)	RUL (bp)	A+T (%)
CameSat001-141	8.9588	7.68	141	61.0	CameSat084-141	0.0088	11.67	141	67.4
CameSat002-187	2.4143	7.18	187	58.8	CameSat085-625	0.0087	2.28	625	61.3
CameSat003-10	1.2658	9.60	10	40.0	CameSat086-159	0.0087	12.12	159	66.0
CameSat004-322	0.6512	17.83	322	67.4	CameSat087-171	0.0085	9.58	171	66.7
CameSat005-499	0.3837	11.62	499	57.5	CameSat088-132	0.0081	12.79	132	56.1
CameSat006-2670	0.2748	2.59	2670	56.1	CameSat089-151	0.0080	14.26	151	68.9
CameSat007-313	0.2600	11.97	313	62.6	CameSat090-288	0.0079	6.61	288	63.2
CameSat008-454	0.2310	8.36	454	60.4	CameSat091-143	0.0079	9.77	143	44.1
CameSat009-206	0.1666	3.63	206	61.2	CameSat092-160	0.0078	13.12	160	68.8
CameSat010-3664	0.1401	3.63	3664	65.9	CameSat093-182	0.0073	16.57	182	63.7
CameSat011-2186	0.1381	8.18	2186	66.3	CameSat094-142	0.0071	7.69	142	62.7
CameSat012-346	0.1372	10.03	346	54.0	CameSat095-231	0.0067	13.07	231	61.5
CameSat013-1223	0.1284	11.99	1223	63.2	CameSat096-135	0.0066	17.45	135	63.0
CameSat014-163	0.1227	16.32	163	58.3	CameSat097-76	0.0066	11.95	76	51.9
CameSat015-288	0.1225	14.10	288	58.8	CameSat098-1148	0.0065	2.57	1148	61.1
CameSat016-449	0.1132	6.45	449	57.5	CameSat099-146	0.0062	10.12	146	61.0
CameSat017-479	0.1115	9.50	479	60.1	CameSat100-146	0.0062	10.34	146	71.2
CameSat018-280	0.0978	10.52	280	67.1	CameSat101-649	0.0061	2.99	649	62.4
CameSat019-228	0.0967	19.91	228	66.2	CameSat102-141	0.0059	11.29	141	66.0
CameSat020-336	0.0665	6.27	336	59.2	CameSat103-172	0.0059	14.49	172	63.4
CameSat021-312	0.0663	13.13	312	62.5	CameSat104-165	0.0058	16.43	165	64.8
CameSat022-324	0.0612	17.54	324	66.7	CameSat105-952	0.0055	2.13	952	63.8
CameSat023-146	0.0571	7.12	146	63.0	CameSat106-309	0.0055	7.04	309	58.3
CameSat024-316	0.0544	4.70	316	54.4	CameSat107-212	0.0053	4.39	212	64.6
CameSat025-150	0.0517	3.75	150	64.7	CameSat108-410	0.0052	18.03	410	65.4
CameSat026-65	0.0514	6.85	65	80.0	CameSat109-148	0.0052	11.11	148	65.5
CameSat027-469	0.0496	5.69	469	62.3	CameSat110-145	0.0052	13.30	145	71.0
CameSat028-154	0.0493	7.78	154	66.2	CameSat111-160	0.0049	10.38	160	64.4
CameSat029-325	0.0461	10.47	325	68.1	CameSat112-147	0.0049	2.93	147	63.9
CameSat030-132	0.0452	7.28	132	63.6	CameSat113-76	0.0048	11.48	76	69.7
CameSat031-281	0.0440	26.18	281	66.9	CameSat114-144	0.0045	15.56	144	62.5
CameSat032-166	0.0434	16.03	166	35.5	CameSat115-14	0.0045	6.70	14	50.0
CameSat033-351	0.0429	16.55	351	60.4	CameSat116-29	0.0044	8.17	29	51.7
CameSat034-191	0.0416	4.64	191	60.7	CameSat117-149	0.0044	8.64	149	67.1
CameSat035-5-tel	0.0414	0.83	5	40.0	CameSat118-144	0.0042	7.07	144	66.0
CameSat036-144	0.0409	4.64	144	66.0	CameSat119-140	0.0040	7.42	140	60.7
CameSat037-1278	0.0405	2.77	1278	64.1	CameSat120-286	0.0040	5.94	286	60.1
CameSat038-50	0.0404	13.65	50	66.0	CameSat121-162	0.0039	18.95	162	71.0
CameSat039-400	0.0389	2.39	400	66.4	CameSat122-132	0.0038	14.77	132	58.3
CameSat040-67	0.0377	13.60	67	64.2	CameSat123-161	0.0038	14.31	161	65.8
CameSat041-142	0.0353	4.81	142	62.7	CameSat124-120	0.0037	15.51	120	59.2
CameSat042-250	0.0342	18.25	250	66.8	CameSat125-309	0.0036	15.66	309	64.4
CameSat043-151	0.0333	5.93	151	65.6	CameSat126-509	0.0036	17.22	509	69.7
CameSat044-2844	0.0322	11.06	2844	73.6	CameSat127-410	0.0034	10.08	410	66.1
CameSat045-143	0.0319	9.01	143	61.5	CameSat128-148	0.0034	6.09	148	57.4
CameSat046-23	0.0301	6.47	23	56.5	CameSat129-91	0.0034	19.47	91	62.6
CameSat047-389	0.0298	13.10	389	64.0	CameSat130-147	0.0033	11.08	147	61.2
CameSat048-293	0.0286	17.01	293	58.4	CameSat131-149	0.0033	12.11	149	70.5
CameSat049-20	0.0265	13.40	20	60.0	CameSat132-429	0.0030	3.43	429	63.4
CameSat050-140	0.0258	9.00	140	67.9	CameSat133-142	0.0028	6.41	142	62.7
CameSat051-3051	0.0255	8.78	3051	67.5	CameSat134-166	0.0028	5.63	166	69.3
CameSat052-143	0.0238	6.67	143	66.4	CameSat135-148	0.0027	7.01	148	66.2
CameSat053-153	0.0235	9.79	153	68.6	CameSat136-255	0.0027	12.48	255	65.9
CameSat054-164	0.0211	8.20	164	67.1	CameSat137-360	0.0025	3.35	360	59.4
CameSat055-30	0.0201	13.58	30	63.3	CameSat138-156	0.0025	7.18	156	57.7
CameSat056-42	0.0198	19.82	42	69.0	CameSat139-369	0.0025	13.38	369	65.0
CameSat057-147	0.0195	9.61	147	61.9	CameSat140-84	0.0025	10.28	84	57.1
CameSat058-280	0.0189	6.36	280	57.1	CameSat141-163	0.0019	13.47	163	65.0
CameSat059-128	0.0188	19.29	128	68.8	CameSat142-408	0.0017	10.77	408	67.9
CameSat060-141	0.0188	14.68	141	59.6	CameSat143-184	0.0016	8.55	184	65.8
CameSat061-230	0.0184	7.56	230	68.3	CameSat144-265	0.0015	13.25	265	63.8
CameSat062-142	0.0183	12.19	142	60.6	CameSat145-168	0.0015	7.16	168	64.3
CameSat063-2048	0.0182	5.04	2048	64.7	CameSat146-139	0.0015	8.36	139	74.1
CameSat064-104	0.0182	2.78	104	51.0	CameSat147-154	0.0014	13.08	154	73.4
CameSat065-148	0.0175	15.05	148	67.6	CameSat148-468	0.0014	4.06	468	59.2
CameSat066-149	0.0175	6.94	149	66.4	CameSat149-141	0.0014	10.48	141	51.8
CameSat067-168	0.0155	23.71	168	69.0	CameSat150-147	0.0013	4.43	147	70.1
CameSat068-153	0.0147	8.35	153	58.8	CameSat151-300	0.0013	7.26	300	63.0
CameSat069-142	0.0144	13.63	142	55.6	CameSat152-228	0.0012	5.41	228	59.6
CameSat070-13	0.0142	14.35	13	53.8	CameSat153-498	0.0012	4.75	498	60.8
CameSat071-20	0.0137	17.80	20	70.0	CameSat154-59	0.0012	9.39	59	72.9
CameSat072-193	0.0132	8.71	193	65.3	CameSat155-496	0.0011	2.09	496	66.7
CameSat073-20	0.0130	22.31	20	45.0	CameSat156-216	0.0011	2.84	216	60.2
CameSat074-1625	0.0116	1.77	1625	63.1	CameSat157-476	0.0011	2.76	476	62.8
CameSat075-275	0.0116	7.13	275	62.9	CameSat158-143	0.0011	9.02	143	65.0
CameSat076-243	0.0114	12.69	243	61.3	CameSat159-272	0.0010	2.51	272	63.6
CameSat077-1817	0.0103	1.95	1817	63.1	CameSat160-36	0.0009	12.37	36	63.9
CameSat078-138	0.0102	14.13	138	67.4	CameSat161-127	0.0009	2.42	127	65.4
CameSat079-143	0.0102	6.60	143	69.2	CameSat162-133	0.0008	4.60	133	70.7
CameSat080-162	0.0098	16.90	162	67.9	CameSat163-416	0.0008	3.37	416	60.6
CameSat081-125	0.0093	16.48	125	58.4	CameSat164-112	0.0006	8.95	112	67.9
CameSat082-204	0.0091	4.98	204	57.8	CameSat165-105	0.0002	8.18	105	60.0
CameSat083-100	0.0091	15.98	100	56.4					

## Data Availability

The data presented in the study are available in the article. The CHRISMAPP script is also available in the GitHub repository (https://github.com/LoriteLab/CHRISMAPP, accessed on 20 February 2024).

## Supplementary Materials

The following are available online at https://www.mdpi.com/article/10.3390/genes15040395/s1, Figure S1: Graphical summary of the clustering results obtained with RepeatExplorer2, displaying the total number of reads used and the repetitive DNA clusters; Figure S2: Alignment between the consensus sequence of the CameSat002-187 satDNA family and the consensus sequence of the CAMA satDNA family, as described by [42]; Figure S3: FISH with (TTAGG)n telomeric probe (red signals) on mitotic chromosomes (counterstained with DAPI in blue) of *C. americana*; Figure S4: Distribution of satellite DNA families in *C. americana* based on the size of their repeat unit; Figure S5: Satellite DNA landscapes for all satDNA families found in *C. americana*; and Figure S6: *C. americana* pseudochromosomes showing the distribution of the main satDNA families obtained through the CHRISMAPP approach; CHRISMAPP script.
