# Supplementary material for: Heterochromatin Is Not the Only Place for satDNAs: The High Diversity of satDNAs in the Euchromatin of the Beetle Chrysolina americana (Coleoptera, Chrysomelidae)
Source: Genes (Basel). 2024 Mar 22;15(4):395. doi: 10.3390/genes15040395 (PMC11049206; doi:10.3390/genes15040395)
Supplement: Supplementary file 1 [file genes-15-00395-s001.zip › Supplementary Figures.pdf]

Supplementary Figures

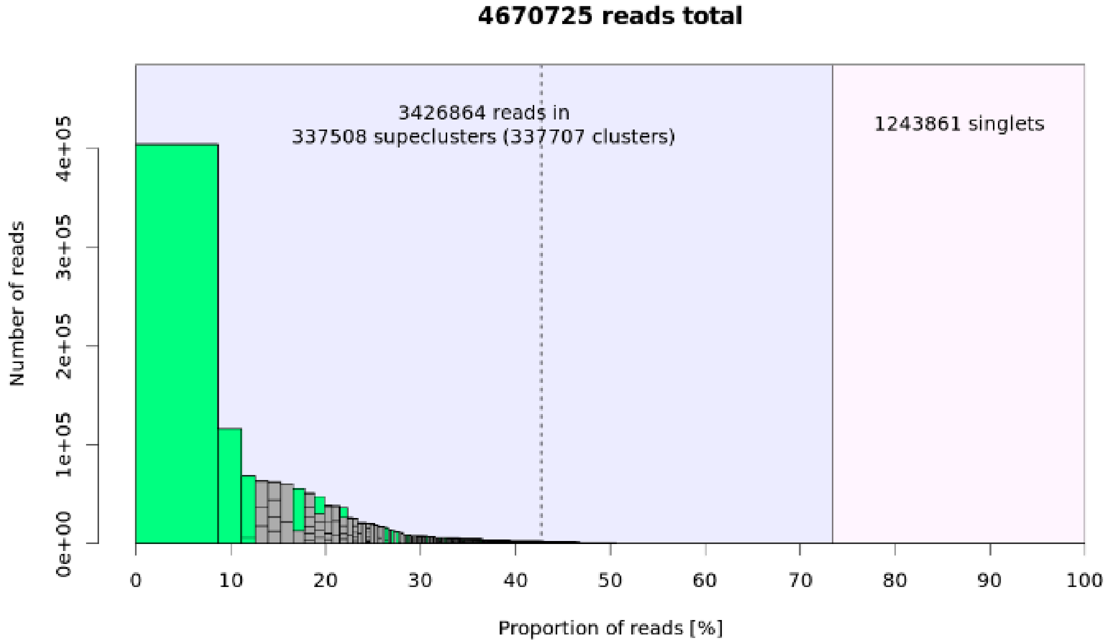

Figure S1. Graphical summary of the clustering results obtained with RepeatExplorer2, displaying the total number of reads used and the repetitive DNA clusters. The clusters representing more than 0.001% of the genome are located to the left of the dotted line. Bars represent superclusters, with their heights and widths corresponding to the numbers of reads in the superclusters (y-axis) and to their proportions in all analyzed reads (x-axis), respectively. Rectangles inside the supercluster bars represent individual clusters. Superclusters containing abundant satellites, as identified by RepeatExplorer, are highlighted in green.

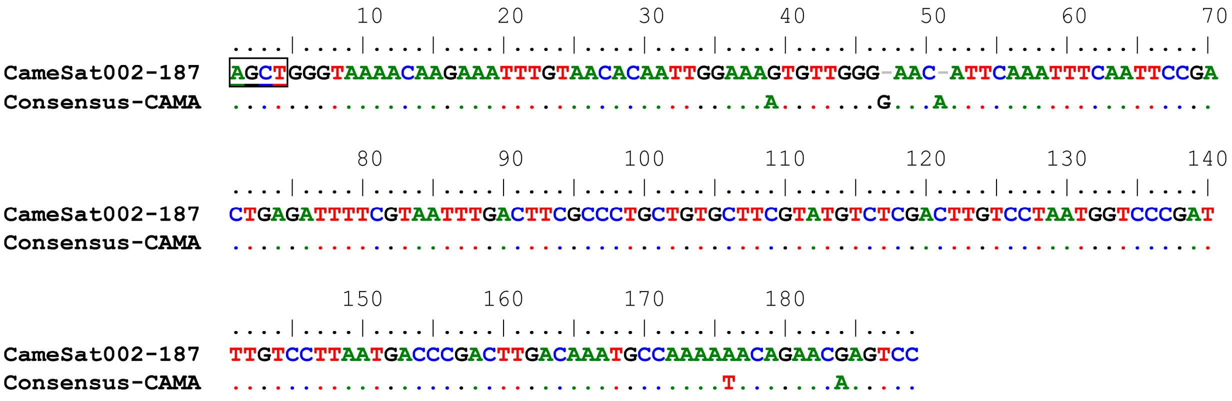

Figure S2. Alignment between the consensus sequence of the CameSat002-187 satDNA family and the consensus sequence of the CAMA satDNA family as described by Lorite *et al.* (2001). The alignment was performed by digesting the sequences with *Alu*I restriction enzymes. In the presented sequence, the box denotes the specific target sequence for this enzyme (AGCT).

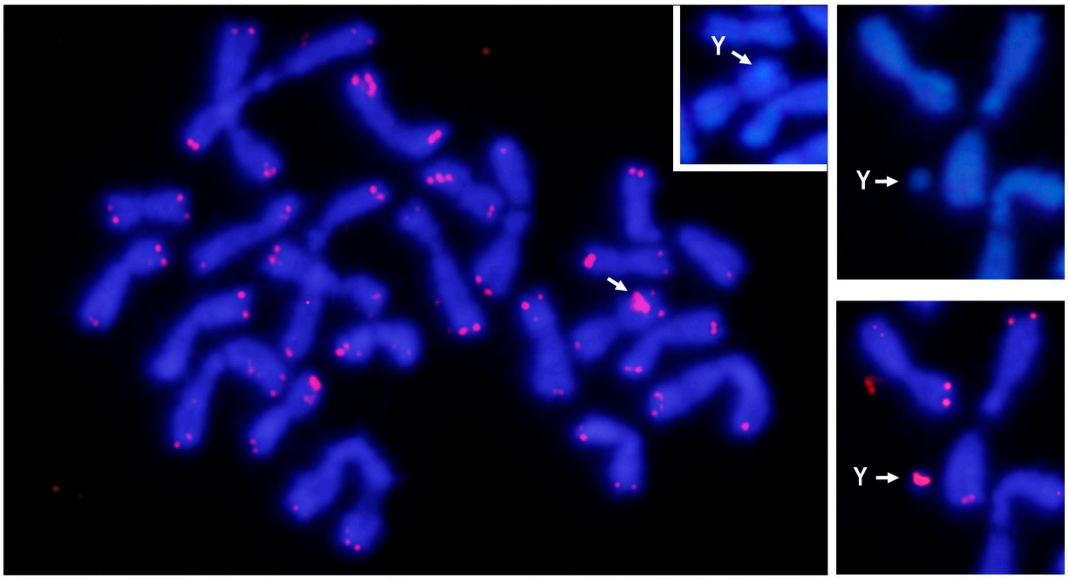

Figure S3. FISH with (TTAGG)n telomeric probe (red signals) on mitotic chromosomes (counterstained with DAPI in blue) of *Chrysolina americana*.

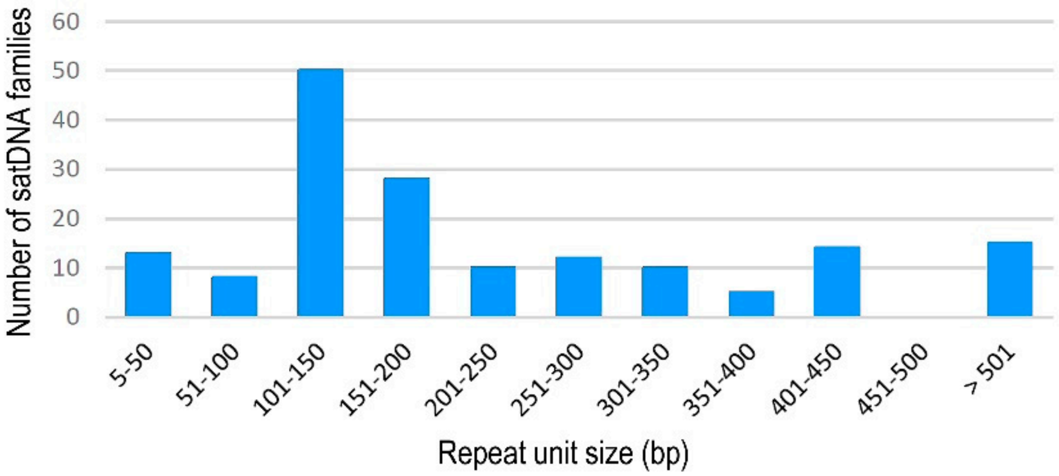

Figure S4. Distribution of satellite DNA families in *Chrysolina americana* based on the size of their repeat unit.

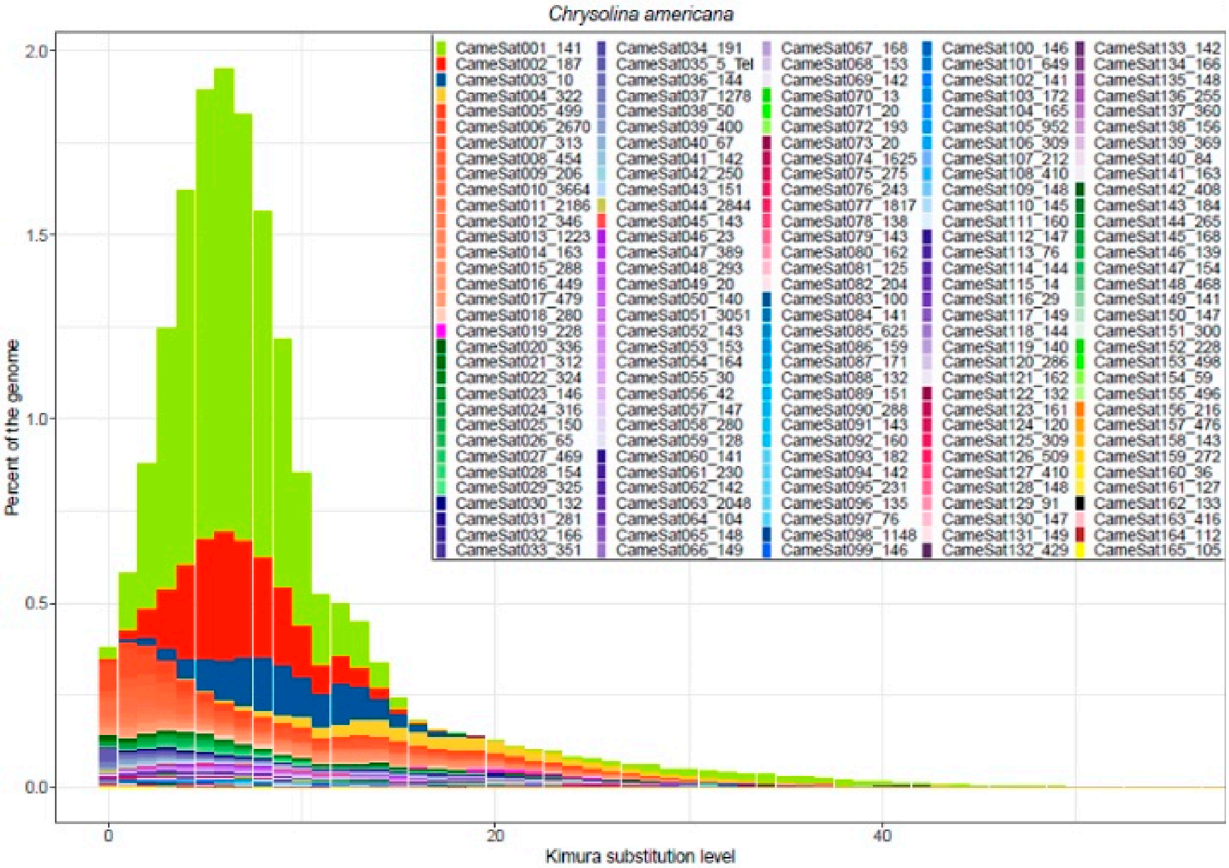
Chrysolina americana
Percent of the genome
Kimura substitution level
CameSat001_141
CameSat002_187
CameSat003_10
CameSat004_322
CameSat005_499
CameSat006_2670
CameSat007_313
CameSat008_454
CameSat009_206
CameSat010_3664
CameSat011_2186
CameSat012_346
CameSat013_1223
CameSat014_163
CameSat015_288
CameSat016_449
CameSat017_479
CameSat018_280
CameSat019_228
CameSat020_336
CameSat021_312
CameSat022_324
CameSat023_146
CameSat024_316
CameSat025_150
CameSat026_65
CameSat027_469
CameSat028_154
CameSat029_325
CameSat030_132
CameSat031_281
CameSat032_166
CameSat033_351
CameSat034_191
CameSat035_5_Tel
CameSat036_144
CameSat037_1278
CameSat038_50
CameSat039_400
CameSat040_67
CameSat041_142
CameSat042_250
CameSat043_151
CameSat044_2844
CameSat045_143
CameSat046_23
CameSat047_389
CameSat048_293
CameSat049_20
CameSat050_140
CameSat051_3051
CameSat052_143
CameSat053_153
CameSat054_164
CameSat055_30
CameSat056_42
CameSat057_147
CameSat058_280
CameSat059_128
CameSat060_141
CameSat061_230
CameSat062_142
CameSat063_2048
CameSat064_104
CameSat065_148
CameSat066_149
CameSat067_168
CameSat068_153
CameSat069_142
CameSat070_13
CameSat071_20
CameSat072_193
CameSat073_20
CameSat074_1625
CameSat075_275
CameSat076_243
CameSat077_1817
CameSat078_138
CameSat079_143
CameSat080_162
CameSat081_125
CameSat082_204
CameSat083_100
CameSat084_141
CameSat085_625
CameSat086_159
CameSat087_171
CameSat088_132
CameSat089_151
CameSat090_288
CameSat091_143
CameSat092_160
CameSat093_182
CameSat094_142
CameSat095_231
CameSat096_135
CameSat097_76
CameSat098_1148
CameSat099_146
CameSat100_146
CameSat101_649
CameSat102_141
CameSat103_172
CameSat104_165
CameSat105_952
CameSat106_309
CameSat107_212
CameSat108_410
CameSat109_148
CameSat110_145
CameSat111_160
CameSat112_147
CameSat113_76
CameSat114_144
CameSat115_14
CameSat116_29
CameSat117_149
CameSat118_144
CameSat119_140
CameSat120_286
CameSat121_162
CameSat122_132
CameSat123_161
CameSat124_120
CameSat125_309
CameSat126_509
CameSat127_410
CameSat128_148
CameSat129_91
CameSat130_147
CameSat131_149
CameSat132_429
CameSat133_142
CameSat134_166
CameSat135_148
CameSat136_255
CameSat137_360
CameSat138_156
CameSat139_369
CameSat140_84
CameSat141_163
CameSat142_408
CameSat143_184
CameSat144_265
CameSat145_168
CameSat146_139
CameSat147_154
CameSat148_468
CameSat149_141
CameSat150_147
CameSat151_300
CameSat152_228
CameSat153_498
CameSat154_59
CameSat155_496
CameSat156_216
CameSat157_476
CameSat158_143
CameSat159_272
CameSat160_36
CameSat161_127
CameSat162_133
CameSat163_416
CameSat164_112
CameSat165_105

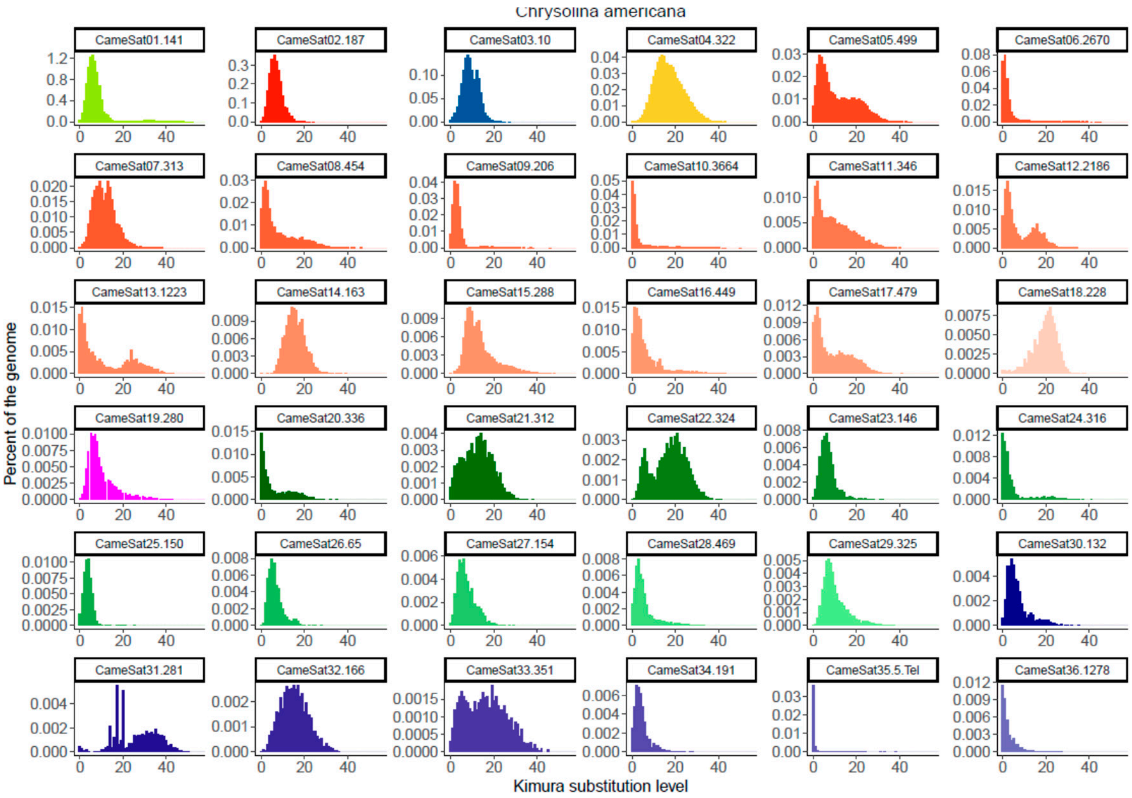
Chrysolina americana
Percent of the genome
Kimura substitution level
CameSat01.141
CameSat02.187
CameSat03.10
CameSat04.322
CameSat05.499
CameSat06.2670
CameSat07.313
CameSat08.454
CameSat09.206
CameSat10.3664
CameSat11.346
CameSat12.2186
CameSat13.1223
CameSat14.163
CameSat15.288
CameSat16.449
CameSat17.479
CameSat18.228
CameSat19.280
CameSat20.336
CameSat21.312
CameSat22.324
CameSat23.146
CameSat24.316
CameSat25.150
CameSat26.65
CameSat27.154
CameSat28.469
CameSat29.325
CameSat30.132
CameSat31.281
CameSat32.166
CameSat33.351
CameSat34.191
CameSat35.5.Tel
CameSat36.1278

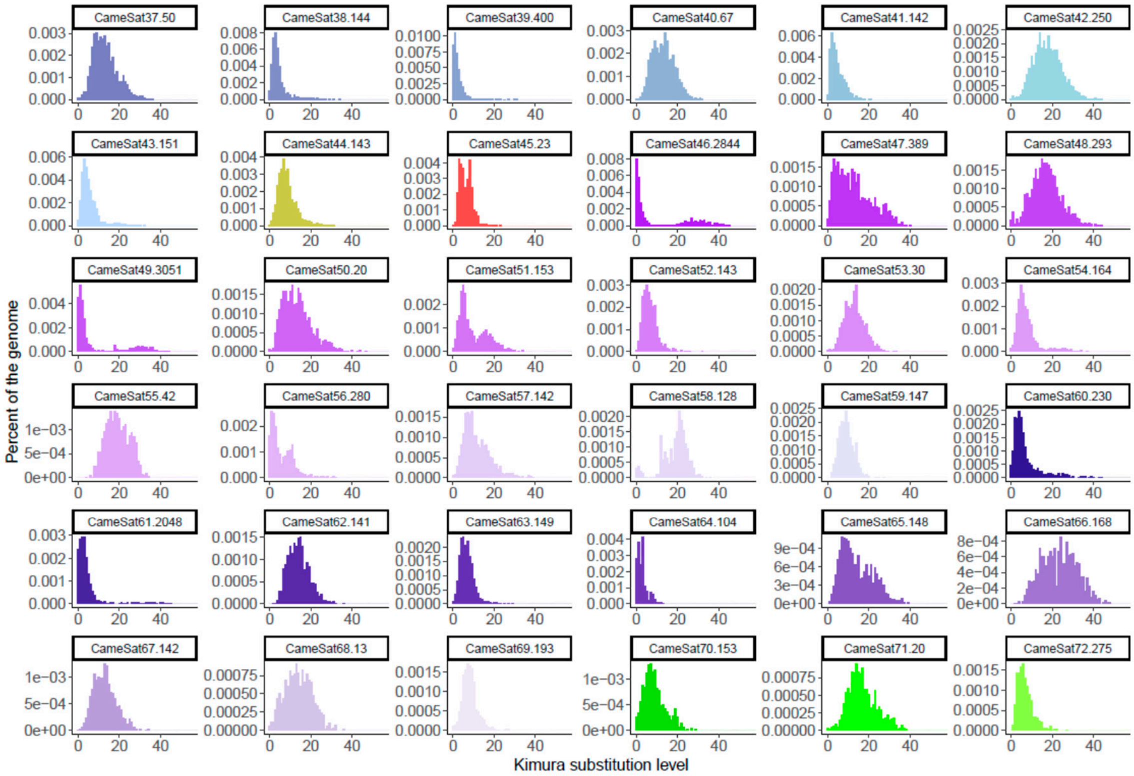
CameSat37.50
CameSat38.144
CameSat39.400
CameSat40.67
CameSat41.142
CameSat42.250
CameSat43.151
CameSat44.143
CameSat45.23
CameSat46.2844
CameSat47.389
CameSat48.293
CameSat49.3051
CameSat50.20
CameSat51.153
CameSat52.143
CameSat53.30
CameSat54.164
CameSat55.42
CameSat56.280
CameSat57.142
CameSat58.128
CameSat59.147
CameSat60.230
CameSat61.2048
CameSat62.141
CameSat63.149
CameSat64.104
CameSat65.148
CameSat66.168
CameSat67.142
CameSat68.13
CameSat69.193
CameSat70.153
CameSat71.20
CameSat72.275
Percent of the genome
Kimura substitution level

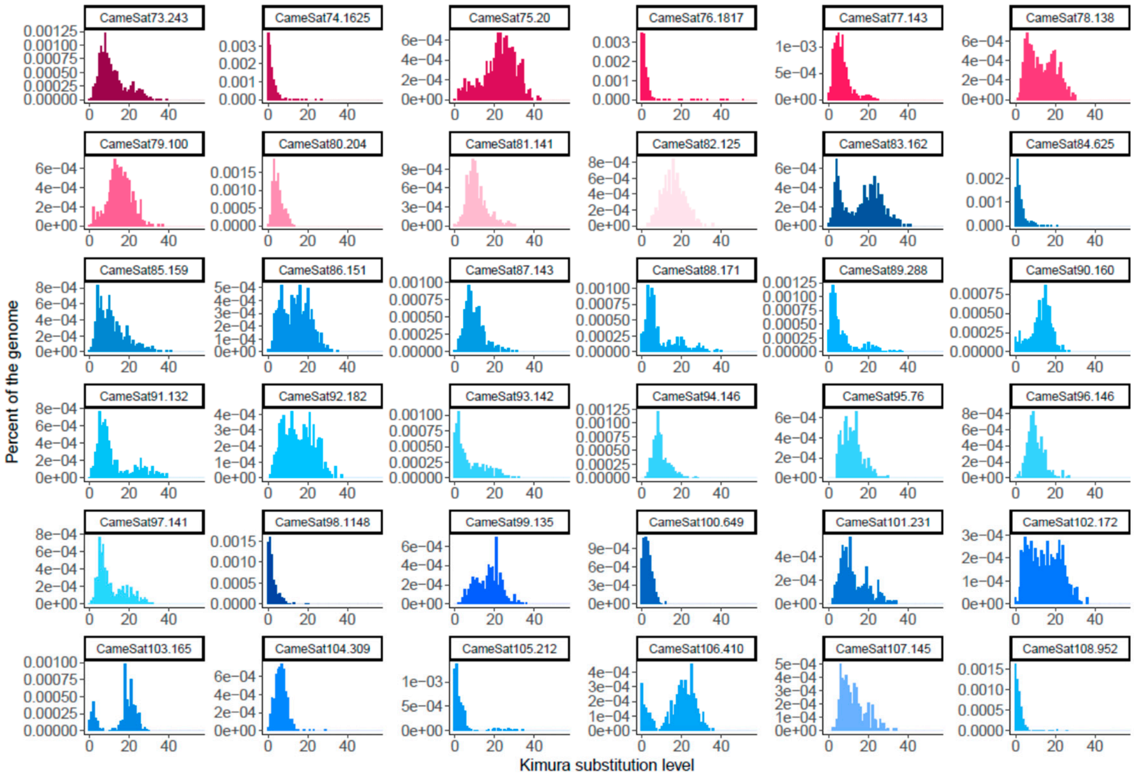
CameSat73.243
CameSat74.1625
CameSat75.20
CameSat76.1817
CameSat77.143
CameSat78.138
CameSat79.100
CameSat80.204
CameSat81.141
CameSat82.125
CameSat83.162
CameSat84.625
CameSat85.159
CameSat86.151
CameSat87.143
CameSat88.171
CameSat89.288
CameSat90.160
CameSat91.132
CameSat92.182
CameSat93.142
CameSat94.146
CameSat95.76
CameSat96.146
CameSat97.141
CameSat98.1148
CameSat99.135
CameSat100.649
CameSat101.231
CameSat102.172
CameSat103.165
CameSat104.309
CameSat105.212
CameSat106.410
CameSat107.145
CameSat108.952
Percent of the genome
Kimura substitution level

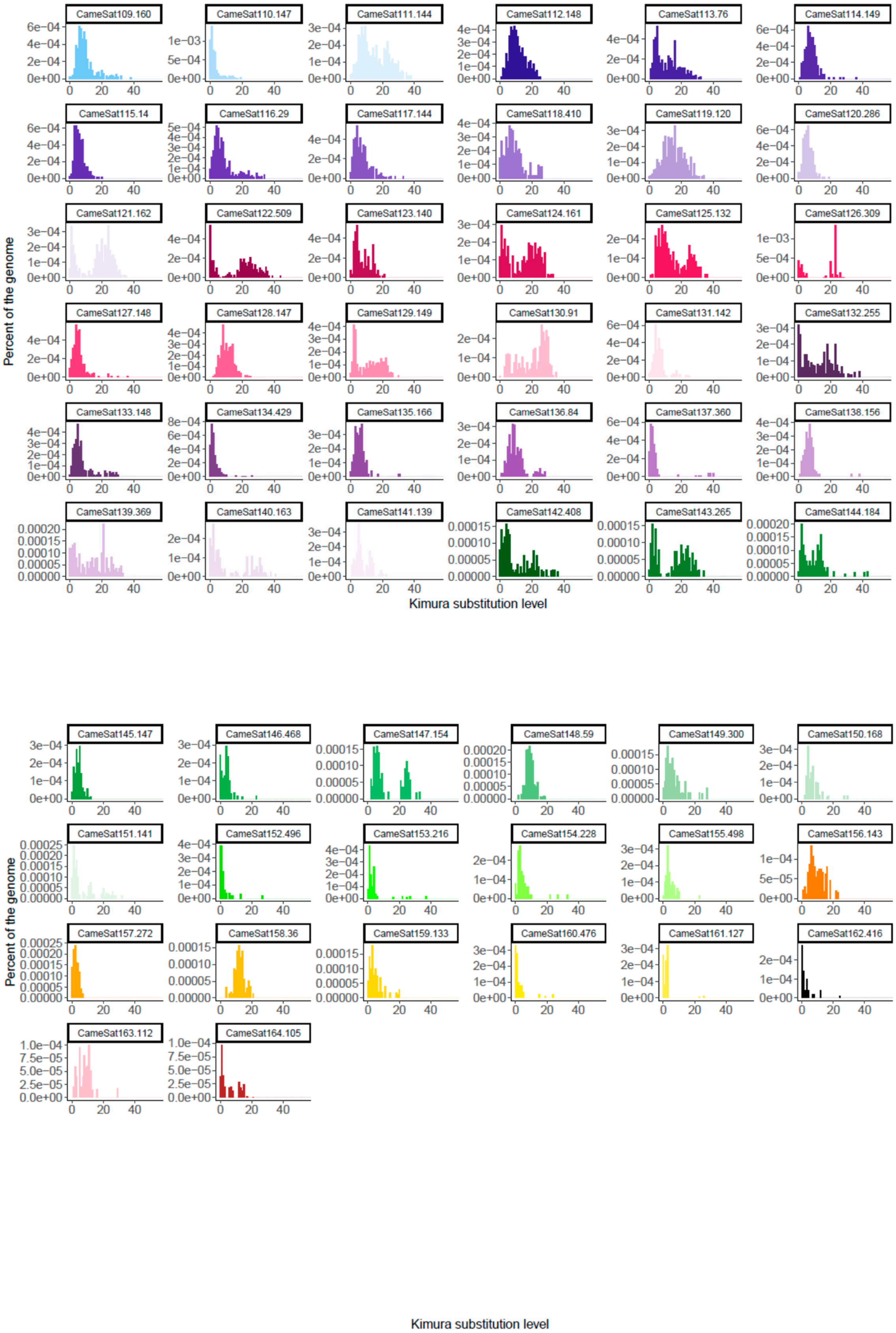

Figure S5. Satellite DNA landscapes for all satDNA families found in *Chrysolina americana.*

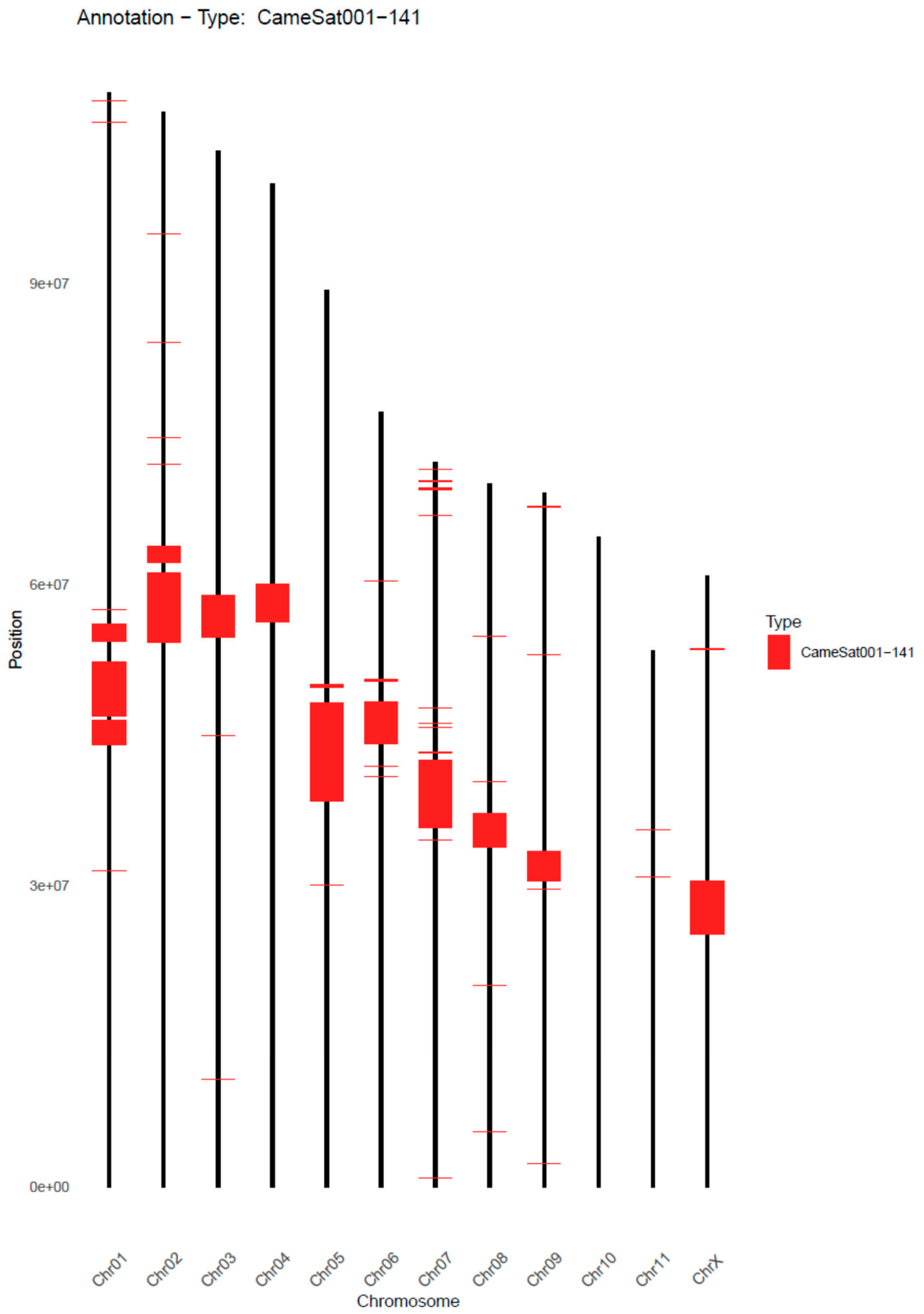
Annotation − Type: CameSat001−141
Position
9e+07
6e+07
3e+07
0e+00
Chr01
Chr02
Chr03
Chr04
Chr05
Chr06
Chr07
Chr08
Chr09
Chr10
Chr11
ChrX
Chromosome
Type
CameSat001−141

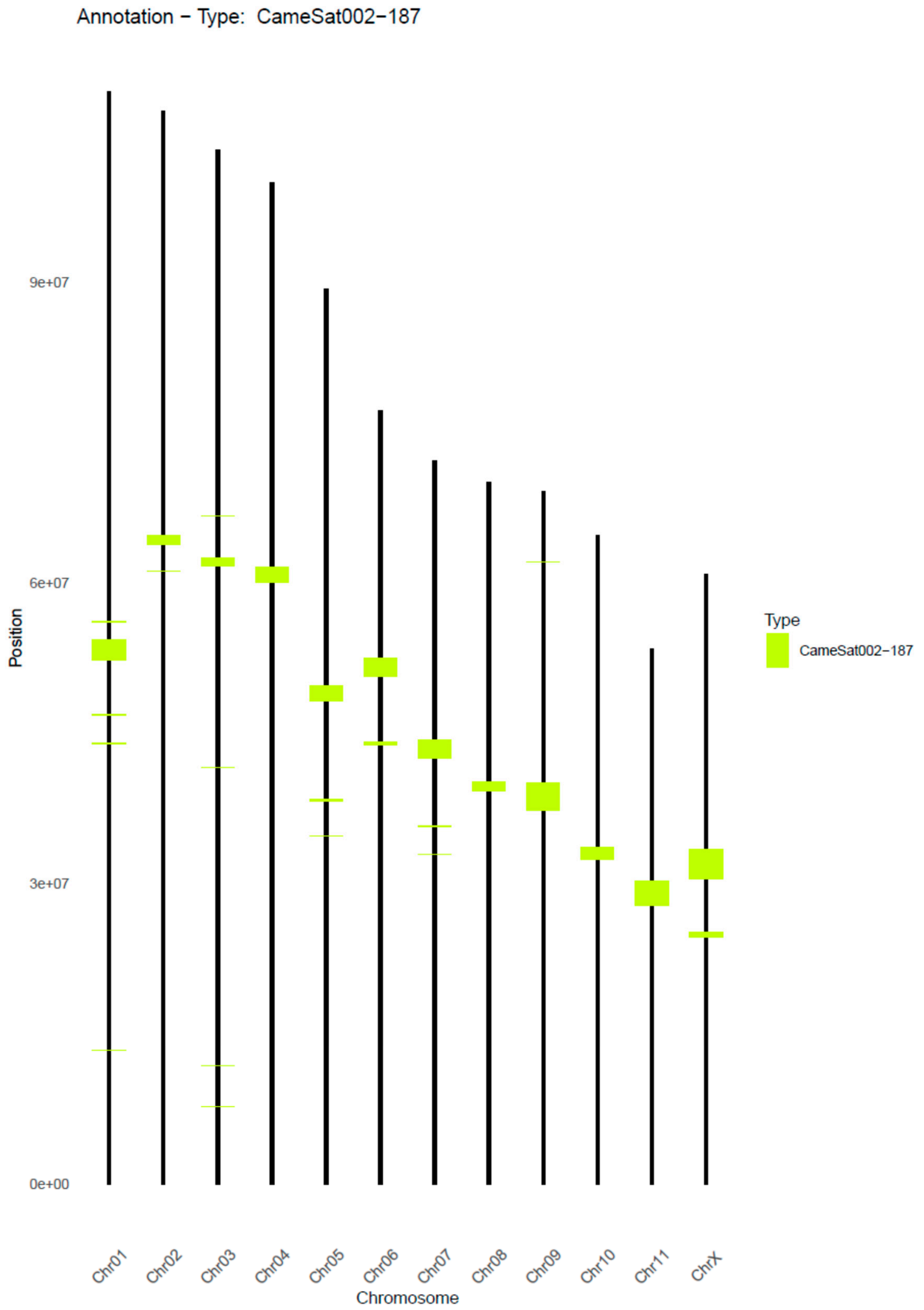

Annotation − Type: CameSat002−187
Position
9e+07
6e+07
3e+07
0e+00
Chr01
Chr02
Chr03
Chr04
Chr05
Chr06
Chr07
Chr08
Chr09
Chr10
Chr11
ChrX
Chromosome
Type
CameSat002−187

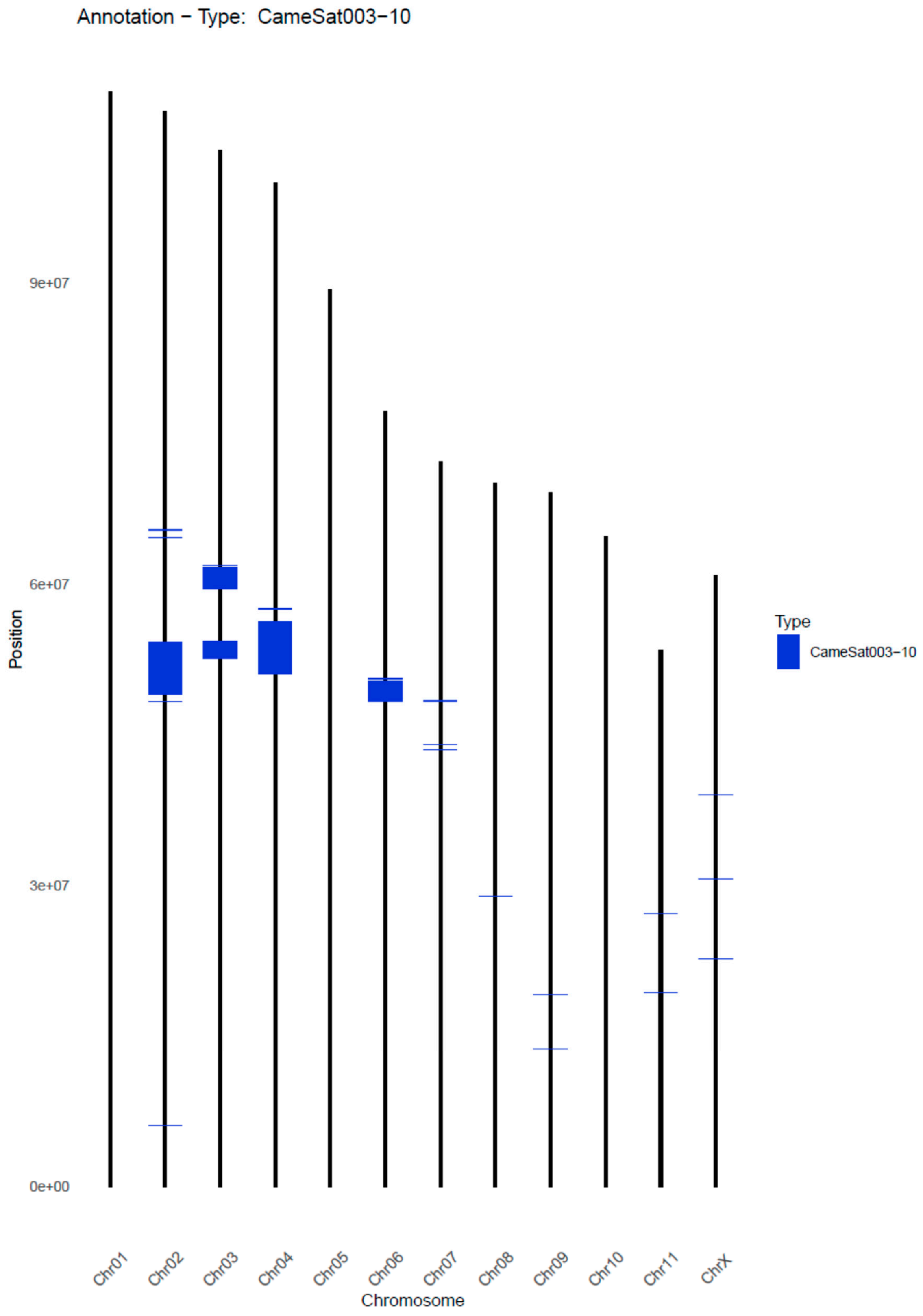
Annotation − Type: CameSat003−10
Position
9e+07
6e+07
3e+07
0e+00
Chr01
Chr02
Chr03
Chr04
Chr05
Chr06
Chr07
Chr08
Chr09
Chr10
Chr11
ChrX
Chromosome
Type
CameSat003−10

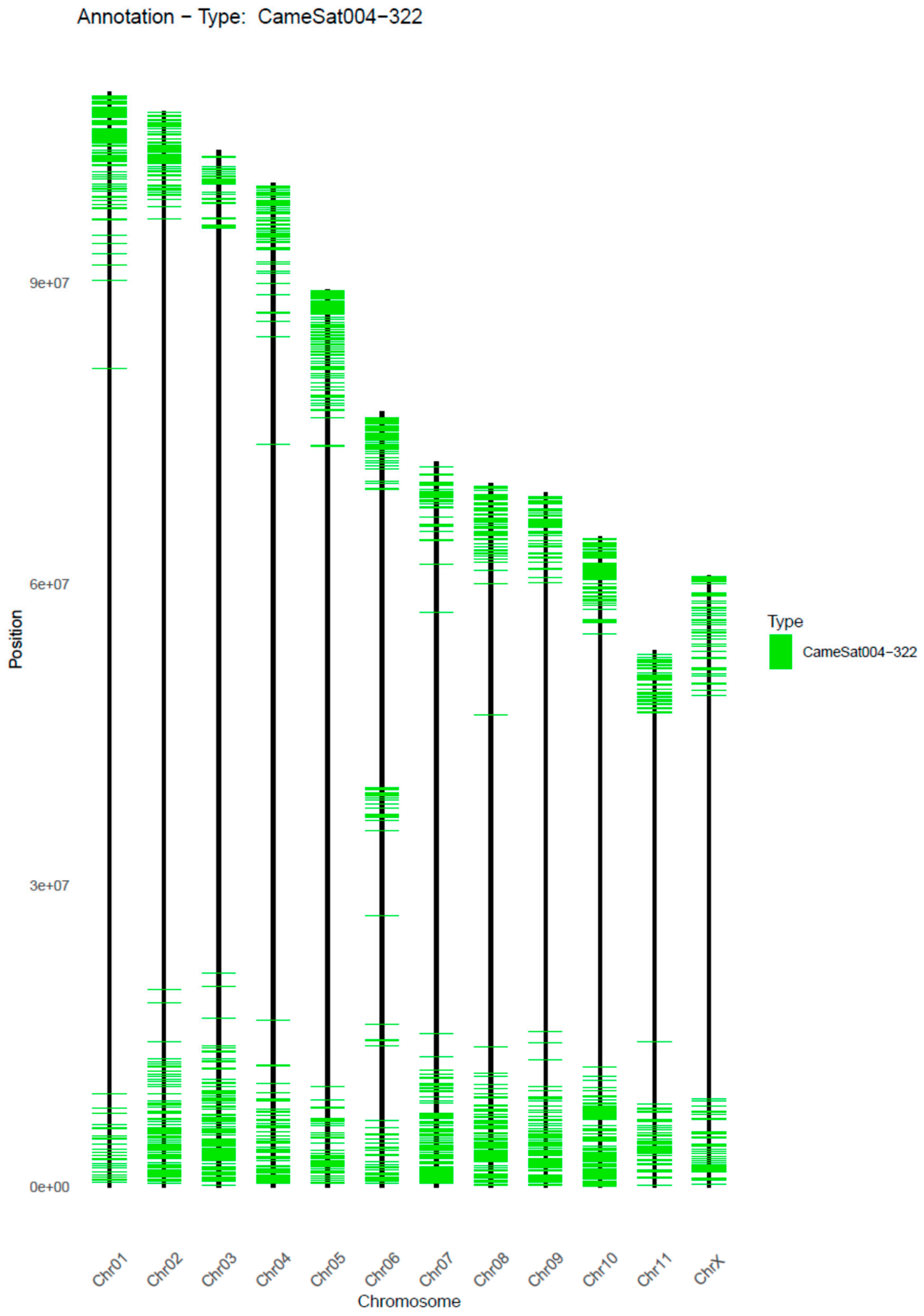

Annotation – Type: CameSat004–322
Position
9e+07
6e+07
3e+07
0e+00
Chr01
Chr02
Chr03
Chr04
Chr05
Chr06
Chr07
Chr08
Chr09
Chr10
Chr11
ChrX
Chromosome
Type
CameSat004–322

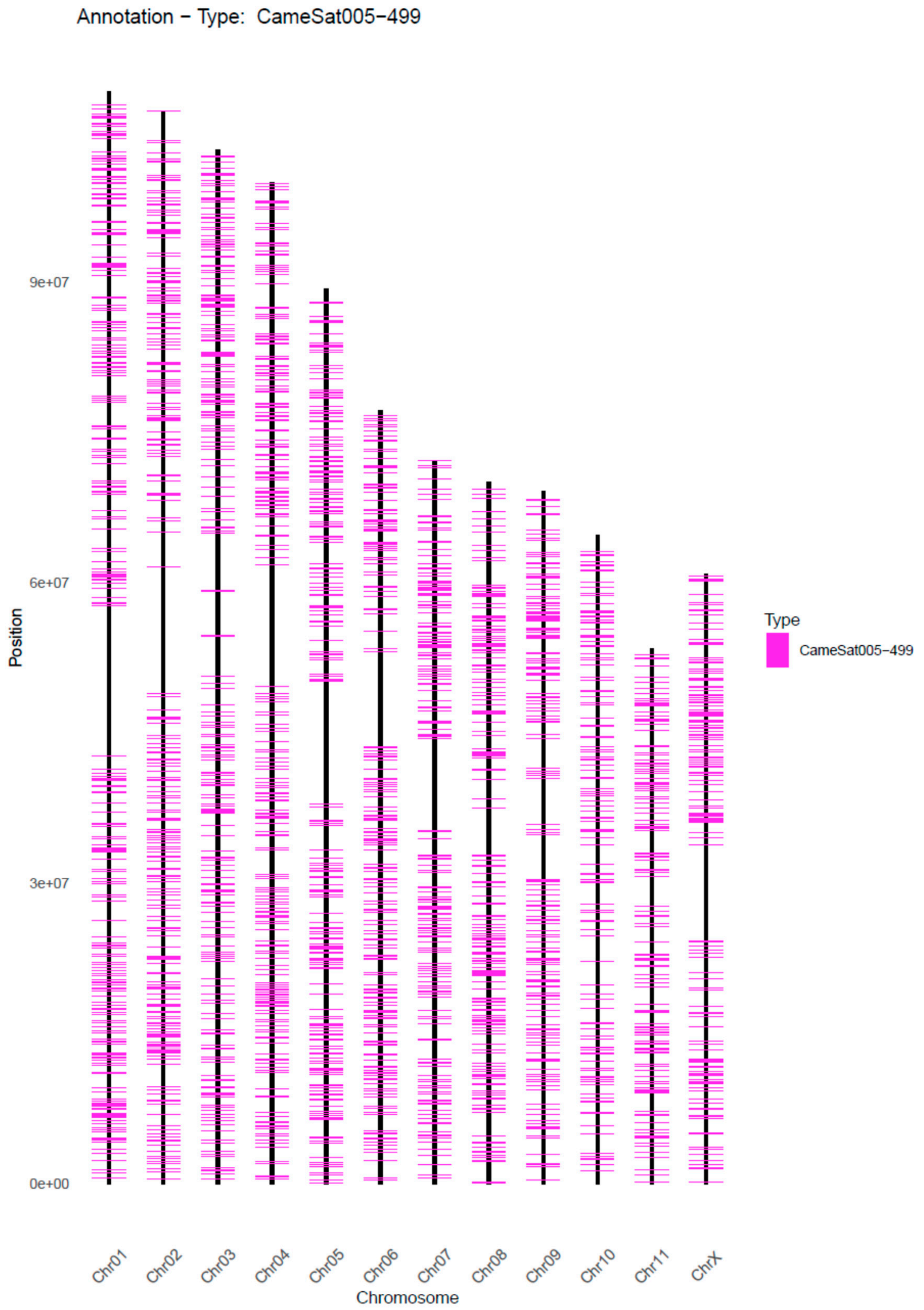

Figure S6. *Chysolina americana* pseudochromosomes showing the distribution of the main satDNA families obtained through the CHRISMAPP approach.
